# Personalised PET imaging in oncology: an umbrella review of meta-analyses to guide the appropriate radiopharmaceutical choice and indication

**DOI:** 10.1007/s00259-024-06882-9

**Published:** 2024-09-11

**Authors:** Margarita Kirienko, Fabrizia Gelardi, Francesco Fiz, Matteo Bauckneht, Gaia Ninatti, Cristiano Pini, Alberto Briganti, Massimo Falconi, Wim J. G. Oyen, Winette T. A. van der Graaf, Martina Sollini

**Affiliations:** 1 Nuclear Medicine, Fondazione IRCCS Istituto Nazionale dei Tumori di Milano, Milan, Italy; 2 Vita-Salute San Raffaele University, Via Olgettina 58, Milan, 20132 Italy; 3 Department of Nuclear Medicine, E.O. “Ospedali Galliera”, Genoa, Italy; 4 Department of Nuclear Medicine and Clinical Molecular Imaging, University Hospital, Tübingen, Germany; 5 Department of Health Science (DISSAL), University of Genoa, Genoa, Italy; 6 Nuclear Medicine, IRCCS Ospedale Policlinico San Martino, Genoa, Italy; 7 School of Medicine and Surgery, University of Milano-Bicocca, Monza, Italy; 8 Department of Nuclear Medicine, IRCCS Ospedale San Raffaele, Milan, 20132 Italy; 9 Division of Oncology/Unit of Urology, URI, IRCCS Ospedale San Raffaele, Milan, Italy; 10 Pancreatic and Transplant Surgery Unit, San Raffaele Hospital, Vita-Salute University, Milan, Italy; 11 Department of Radiology and Nuclear Medicine, Rijnstate Hospital, Arnhem, The Netherlands; 12 Department of Biomedical Sciences, Humanitas University, Milan, Italy; 13 Department of Nuclear Medicine, Humanitas Clinical and Research Center, Milan, Italy; 14 Department of Medical Oncology, Netherlands Cancer Institute - Antoni van Leeuwenhoek, Amsterdam, The Netherlands; 15 Department of Medical Oncology, Erasmus MC Cancer Institute, Erasmus University Medical Center, Rotterdam, The Netherlands

**Keywords:** Positron emission tomography, PET/CT, PET/MR, Oncologic imaging

## Abstract

**Purpose:**

For several years, oncological positron emission tomography (PET) has developed beyond 2-deoxy-2-[^18^F]fluoro-D-glucose ([^18^F]FDG). This umbrella review of meta-analyses aims to provide up-to-date, comprehensive, high-level evidence to support appropriate referral for a specific radiopharmaceutical PET/computed tomography (CT) or PET/magnetic resonance (MR) in the diagnosis and staging of solid cancers other than brain malignancies.

**Methods:**

We performed a systematic literature search on the PubMed/MEDLINE and EMBASE databases for meta-analyses assessing the accuracy of PET/CT and/or PET/MRI with [^18^F]FDG, somatostatin- receptor-targeting ^68^Ga-DOTA-peptides, ^18^F-labelled dihydroxyphenylalanine ([^18^F]DOPA), prostate-specific membrane antigen (PSMA)-targeted radioligands, and fibroblast activation protein inhibitors (FAPI) in the diagnosis/disease characterisation and staging of solid cancers other than brain tumours.

**Results:**

The literature search yielded 449 scientific articles. After screening titles and abstracts and applying inclusion and exclusion criteria, we selected 173 meta-analyses to assess the strength of evidence. One article was selected from references. Sixty-four meta-analyses were finally considered. The current evidence corroborates the role of [^18^F]FDG as the main player in molecular imaging; PSMA tracers are useful in staging and re-staging prostate cancer; somatostatin-targeting peptides (e.g. [^68^Ga]Ga- DOTA-TOC and -TATE) or [^18^F]DOPA are valuable in neuroendocrine tumours (NETs). FAPI has emerged in gastric cancer assessment. According to search and selection criteria, no satisfactory meta-analysis was selected for the diagnosis/detection of oesophageal cancer, the diagnosis/detection and N staging of small cell lung cancer and hepatic cell carcinoma, the diagnosis/detection and M staging of melanoma and Merkel cell carcinoma, cervical, vulvar and penis cancers, the N and M staging of lung and gastroenteropancreatic NET, testicular cancer, and chondrosarcoma, and the M staging of differentiated thyroid, bladder and anal cancers.

**Conclusion:**

The comprehensive high-level evidence synthesised in the present umbrella review serves as a guiding compass for clinicians and imagers, aiding them in navigating the increasingly intricate seascape of PET examinations.

**Supplementary Information:**

The online version contains supplementary material available at 10.1007/s00259-024-06882-9.

## Introduction

The development of guideline recommendations aims to improve and harmonise patient management. Consensus recommendations are generated through a synthesis of published evidence and clinical experience. Multidisciplinary experts participate and vote on recommendation statements. However, expert panels contributing to cancer-focused guidelines rarely include nuclear medicine specialists [1–6].

Progress in molecular biology, biochemistry, and genetics paved the way for developing increasingly specific radiopharmaceuticals targeting different metabolic pathways, receptors, and cell membrane proteins. While 2-deoxy-2-[^18^F]fluoro-D-glucose ([^18^F]FDG) is still the most used Positron Emission Tomography (PET) radiotracer worldwide, several others have been introduced in routine clinical practice for a more accurate evaluation of various oncological diseases. This trend is expected to grow exponentially in the upcoming years. The most remarkable examples in oncology are radiolabelled somatostatin analogues, ^18^F-labeled dihydroxyphenylalanine ([^18^F]DOPA) [7], prostate-specific membrane antigen (PSMA)-targeted radioligands [8], and radiolabelled fibroblast activation protein (FAP) inhibitors [9].

Moreover, novel radiolabelled tracers are under development and might join the clinical fray in the upcoming years. Finally, some PET tracers did not stand the test of time and are no longer utilised or available. In this dynamic scenario, many professionals outside the nuclear medicine field may encounter increasing difficulties in choosing the most appropriate radiopharmaceutical for a specific clinical setting. On the one hand, this may prevent patients from being offered the best available diagnostic modality for assessing their disease, which may also significantly impact their management and overall oncological outcome. Conversely, inappropriate exam requests may result in the necessity to prescribe other radiological investigations and/or another PET scan with a different radiopharmaceutical, resulting in higher costs and radiation exposure.

This umbrella review of meta-analyses aims to provide up-to-date, comprehensive, high-level evidence to support appropriate referral for a specific radiopharmaceutical PET/CT or PET/MRI for the diagnosis and staging of solid cancers other than brain tumours.

## Materials and methods

The present review was performed following the Preferred Reporting Items for Systematic Reviews and Meta-Analyses (PRISMA) statement [10] and was registered in the International Prospective Register of Systematic Reviews (PROSPERO) with ID: CRD42023414744.

### Search strategy, eligibility criteria, and study selection

We performed a systematic literature search on the PubMed/MEDLINE and EMBASE databases for meta-analyses assessing the role of PET imaging with different radiopharmaceuticals in diagnosing and staging solid tumours other than brain cancer. The search was extended until April 1st, 2023. Details about the search algorithm are shown in Supplementary Materials. Additionally, references were screened to identify other relevant papers.

We included meta-analyses assessing the accuracy of PET/CT and/or PET/MRI with [^18^F]FDG, [^68^Ga]-DOTA-peptides, [^18^F]DOPA, PSMA-targeted radioligands, and FAPI agents in the diagnosis/disease characterisation and staging of patients with rare and non-rare solid cancers other than brain tumours. The following exclusion criteria were applied: (1) systematic review without meta-analysis; (2) clinical setting other than diagnosis/disease characterisation or staging; (3) non-English language; (4) lack of conventional metrics for reporting the diagnostic accuracy results.

Five authors (FF, FG, MB, MK, and MS) independently screened titles and abstracts of meta-analyses obtained after the literature search and assessed their eligibility. In the event of discrepancies between the authors, a second round of screening was performed, and any disagreement was solved by majority voting.

### Data extraction, grading of evidence and statistical analysis

For each selected meta-analysis, we collected the following information in a database: (1) clinical setting (diagnosis, T, N and M staging); (2) study design (prospective vs. retrospective); (3) number of included studies; (4) radiopharmaceutical(s) employed; (5) imaging modality (PET, PET/CT, or PET/MRI); (6) type of analysis (per-lesion, per-patient, mixed); (7) number of included patients (per-patient) and lesions (per-lesions); (8) accuracy metrics with 95% Confidence Intervals (95% CIs) (pooled AUC, sensitivity, specificity, and I^2^ heterogeneity index for both sensitivity and specificity).

Five authors (FF, FG, MB, MK, and MS) independently evaluated the strength of evidence of included meta-analyses by assigning a score from 1 to 4 taking into consideration the following parameters: (1) design of the included studies (1 = only prospective; 0.75 = prospective + retrospective; 0.5 = only retrospective); (2) number of included studies (1 > 5; 0.75 = 3–5; 0.5 = 3); (3) I^2^ heterogeneity index for both sensitivity and specificity (1 = I^2^ < 25%; 0.50 = I^2^ 25–50%; 0.25 = I^2^ 50–75%; 0 = I^2^ > 75%).

Considering the grading of evidence scores, we finally categorised meta-analyses into three classes according to their level of evidence: ≤1 = weak; 1–3 = moderate; ≥3 strong. In case of a lack of available meta-analyses on a specific disease, the score 0 (no evidence) was assigned.

The meta-analyses with the highest scores for each author were selected for each solid tumour type, clinical setting, imaging modality, and radiopharmaceutical. Selected meta-analyses were reviewed, and the score was re-evaluated in a second round to solve any difference between authors. Finally, the meta-analyses with the highest scores were selected as references to generate recommendations. In the event of a tie, the most recent year of publication and the largest number of patients were used as tiebreaker. Accuracy metrics were reported separately in meta-analysis with per-patient and per-lesion sub-analysis. Accordingly, accuracy metrics were reported separately when PET-only or hybrid modality sub-analyses were available. In case no evidence on the widely used PET radiopharmaceuticals could be identified, a potential alternative, which has the potential to advance to evidence-based implementation into clinical practice, is suggested. Excel ^®^ 2017 (Microsoft^®^, Redmond, WA, the United States of America) was used to synthesise included meta-analyses qualitatively.

## Results

The literature search yielded 449 scientific articles. After screening titles and abstracts and applying inclusion and exclusion criteria, we selected 173 meta-analyses to assess the strength of evidence. One article was identified by screening the references. Of these, 33 had weak strength of evidence, 127 moderate, and 14 strong. Sixty-four meta-analyses were finally considered to generate recommendations for each tumour histotype and clinical setting. No meta-analyses passed the selection round for mesothelioma, thymoma, medullary thyroid carcinoma and neuroendocrine carcinoma. The principal reasons for exclusion were as follows: the presence of a heterogeneous cohort of tumour histotypes, the use of metrics other than sensitivity and specificity, and the absence of discrete analyses for TNM stages and between patients and lesions.

**Fig. 1:**
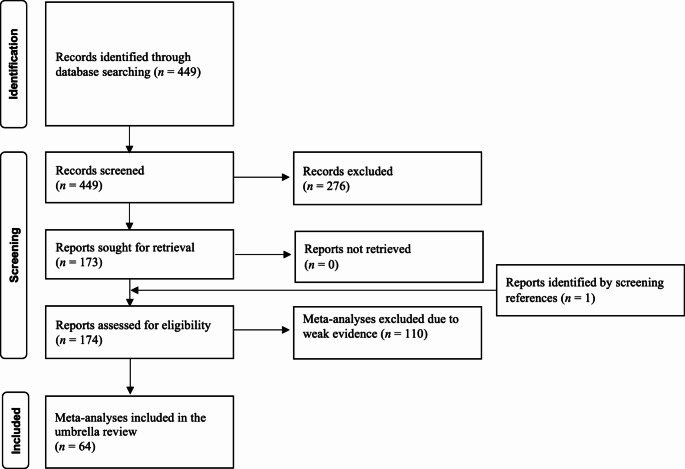
Flowchart summarising the article selection process

**Fig. 2:**
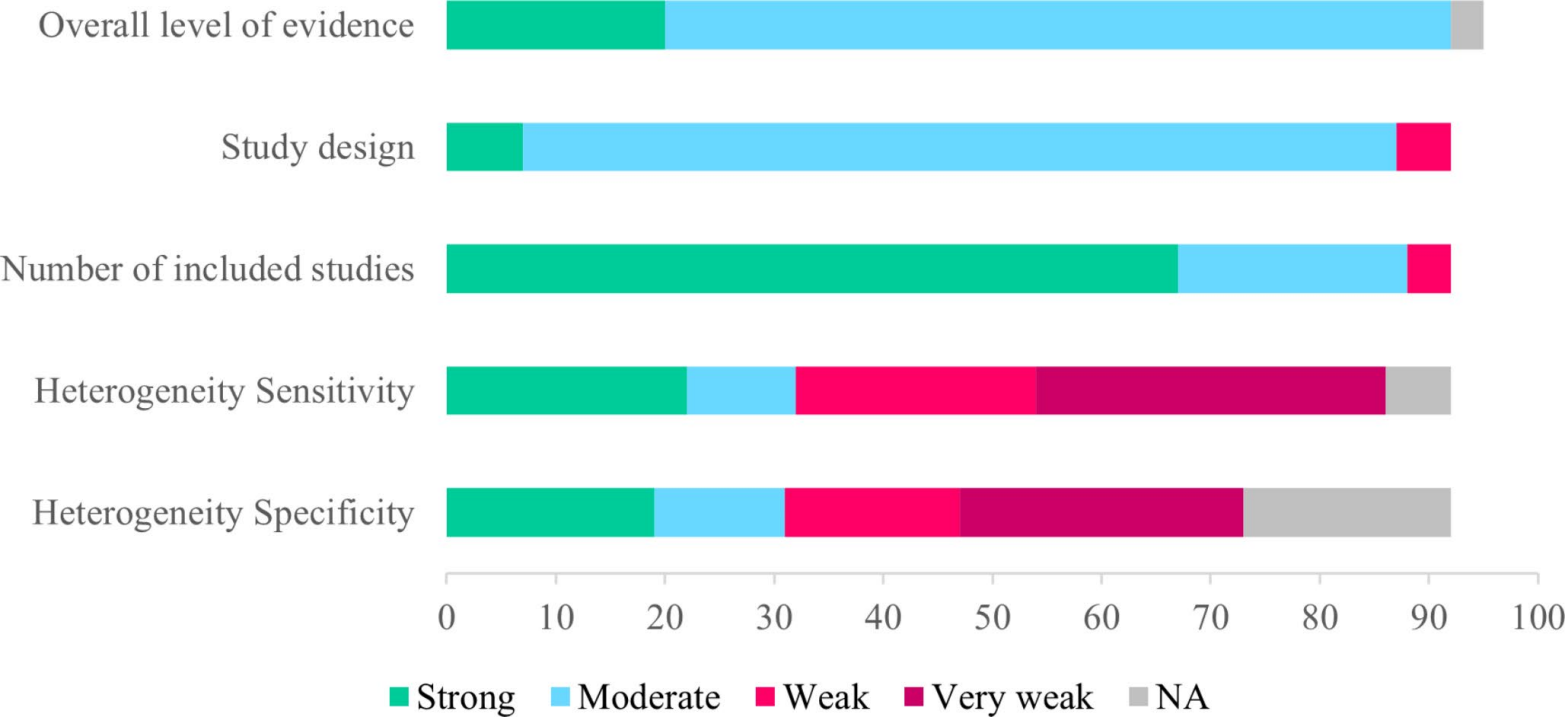
Results of the grading of the strength of evidence of meta-analyses

A flowchart summarising the selection process is shown in Fig. 1. The main results are summarised in Table 1. Figure 2 reports the results of the grading of the strength of evidence. A detailed summary of the results for each clinical setting is provided in Supplementary Table 1.

**Table 1:** Summary of the main findings for each disease and clinical setting

Disease	Setting	Radiopharmaceutical	Technique
*Head and Neck cancer*	*Diagnosis/Detection*	[^18^F]FDG	PET/MRI
	*N and M staging*	[^18^F]FDG	PET/CT
*Non-Small Cell Lung* *Cancer*	*Diagnosis/Detection*	[^18^F]FDG or [^18^F]FLT	PET or PET/CT
	*N and M staging*	[^18^F]FDG	PET/CT
*Small Cell Lung* *Cancer*	*Diagnosis/Detection and N staging*	No evidence*	No evidence*
	*Bone M staging*	[^18^F]FDG	PET or PET/CT
*Lung NET*	*Diagnosis/Detection*	DOTA-peptides	PET/CT
	*N and M staging*	No evidence*	No evidence*
*Insulinoma*	*Diagnosis/Detection*	GLP-1R	PET/CT
*Differentiated thyroid cancer*	*Diagnosis/Detection*	[^18^F]FDG	PET
	*N staging*	[^18^F]FDG	PET/CT
	*M staging*	No evidence*	No evidence*
*Breast cancer*	*Diagnosis/Detection*,* N and M staging*	[^18^F]FDG	PET/MRI
*Oesophageal cancer*	*Diagnosis/Detection*	No evidence*	No evidence*
	*N and M staging*	[^18^F]FDG	PET/CT
*Gastric cancer*	*Diagnosis/Detection*	FAPI	PET/CT and PET/MRI
	*N and M staging*	FAPI	PET/CT and PET/MRI
*Colorectal cancer*	*Diagnosis/Detection*	[^18^F]FDG	PET/MRI
	*N and M staging*	[^18^F]FDG	PET/MRI
*Anal cancer*	*Diagnosis/Detection and N staging*	[^18^F]FDG	PET/CT
	*M staging*	No evidence*	No evidence*
*Pancreatic cancer*	*Diagnosis/Detection*,* N and M staging*	[^18^F]FDG	PET/CT
*Hepatic cell* *carcinoma*	*Diagnosis/Detection and N staging*	No evidence*	No evidence*
	*M staging*	[^18^F]FDG	PET/CT
*Gallbladder cancer*	*Diagnosis/Detection*	[^18^F]FDG	PET or PET/CT
*Cholangiocarcinoma*	*Diagnosis/Detection*,* N and M staging*	[^18^F]FDG	PET or PET/CT
*GEP NET*	*Diagnosis/Detection*	DOTA-peptides	PET or PET/CT
	*N and M staging*	No evidence*	No evidence*
*Insulinoma*	*Diagnosis/Detection*	No evidence*	No evidence*
*Pheochromocytoma and Paraganglioma*	*Diagnosis/Detection*	DOTA-peptides	PET or PET/CT
	*N and M staging*	[^18^F]FDG	PET/CT
*Renal Cell Carcinoma*	*Diagnosis/Detection*,* N and M staging*	[^18^F]FDG	PET/CT
*Bladder cancer*	*Diagnosis/Detection and N staging*	[^18^F]FDG	PET/CT
	*M staging*	No evidence*	No evidence*
*Ovarian cancer*	*Diagnosis/Detection*,* N and M staging*	[^18^F]FDG	PET/CT
*Endometrial cancer*	*Diagnosis/Detection*,* N and M staging*	[^18^F]FDG	PET/CT
*Cervical cancer*	*Diagnosis/Detection and M staging*	No evidence*	No evidence*
	*N staging*	[^18^F]FDG	PET or PET/CT
*Vulvar cancer*	*Diagnosis/Detection and M staging*	No evidence*	No evidence*
	*N staging*	[^18^F]FDG	PET or PET/CT
*Prostate cancer*	*Diagnosis/Detection*	PSMA	PET/CT
	*N staging*	PSMA	PET/MRI
	*M bone staging*	PSMA or choline or [^18^F]NaF	PET/CT
*Testicular cancer*	*Diagnosis/Detection*	[^18^F]FDG	PET/CT
	*N and M staging*	No evidence*	No evidence*
*Penile cancer*	*Diagnosis/Detection and M staging*	No evidence*	No evidence*
	*N staging*	[^18^F]FDG	PET/CT
*Melanoma*	*Diagnosis/Detection and M staging*	No evidence*	No evidence*
	*N staging*	[^18^F]FDG	PET/CT
*Merkel Cell* *Carcinoma*	*Diagnosis/Detection and M staging*	No evidence*	No evidence*
	*N staging*	[^18^F]FDG	PET/CT
*Ewing Sarcoma*	*Diagnosis/Detection*,* N and M lung and bone staging*	[^18^F]FDG	PET or PET/CT
*Chondrosarcoma*	*Diagnosis/Detection*	[^18^F]FDG	PET/CT
	*N and M staging*	No evidence*	No evidence*

CT – computed tomography; [^18^F]FDG – 2-deoxy-2-[^18^F]fluoro-D-glucose; DOTA-peptides – somatostatin receptor targeting PET radiopharmaceuticals with DOTA; FAPI – fibroblast activation protein inhibitors; GEP NET – gastroenteropancreatic neuroendocrine tumours; GLP-1R - Glucagon-like peptide-1 receptor; NET – neuroendocrine tumours; MRI – magnetic resonance imaging; N staging – lymph-nodal staging; M staging – distant metastases staging; PET – positron emission tomography; PSMA – prostate specific membrane antigen

* No evidence – no meta-analyses identified according to search and selection criteria of the present review

### Head and neck

The radiopharmaceutical of choice is [^18^F]FDG for diagnostic purposes. PET/MR imaging modality, on a per-lesion analysis, demonstrated a sensitivity of 0.91 (95%CI 0.89–0.93), a specificity of 0.63 (95%CI 0.60–0.66), and an AUC of 0.96 [11]. Lymph-node staging benefits from [^18^F]FDG PET/CT. Indeed, this modality showed a sensitivity of 0.90 (95%CI 0.87–0.93) and a specificity of 0.92 (95%CI 0.89–0.95) [12]. [^18^F]FDG PET/CT (and PET) is indicated for distant metastases evaluation. On a per-patient analysis, sensitivity resulted in 0.83 (95%CI 0.77–0.88), and specificity 0.97 (95%CI 0.95–0.98) [13].

Summary of evidence & Comments [^18^F]FDG PET/CT is recommended for the initial staging of stage III, IV (T3-4, N1-3) cancers to search for distant metastases [14]. [^18^F]FDG PET/CT can be proposed for patients with disease at any stage to exclude synchronous neoplasms, which might change subsequent treatment [15].

### Non-small cell lung cancer

[^18^F]FDG PET/CT (or PET) scan is useful for lung lesion diagnosis, particularly in differentiating between cancerous and infectious lesions, even in areas with a high prevalence of infectious diseases. On a per-lesion basis, sensitivity and specificity were 0.89 (95%CI 0.86–0.91) and 0.75 (95%CI 0.71–0.79), respectively [16]. The dual time-point and single time-point acquisition demonstrated similar accuracy in the differential diagnosis of pulmonary nodules. However, dual time-point imaging appears more specific than single time-point PET/CT: 73% vs. 59% [17]. Alternatively, [^18^F]FLT can be used for differential diagnosis between malignant and benign lesions; indeed, [^18^F]FLT PET/CT demonstrated a sensitivity and specificity of 0.80 (95%CI 0.74–0.85) and 0.82 (95%CI 0.74–0.88) [18]. [^18^F]FDG PET/CT is indicated for lymph-nodal staging, given its high specificity (0.93, 95%CI 0.93–0.94) [19]. For distant metastases staging, [^18^F]FDG PET/CT yields an AUC of 0.96 (95%CI 0.94–0.97) [20]. For bone metastases detection, in particular, the diagnostic performance of PET/CT resulted as follows: sensitivity 0.92 (95%CI 0.88–0.95), specificity 0.98 (95%CI 0.97–0.98); lower performance was shown for PET alone [21].

### Small cell lung cancer

In Small Cell Lung Cancer (SCLC), published data demonstrated [^18^F]FDG PET or PET/CT usefulness in bone M staging with an AUC, sensitivity and specificity of 0.98, 0.97 (95%CI 0.94–0.99), and 0.98 (95%CI 0.95–0.99), respectively [22].

Summary of evidence & Comments [^18^F]FDG PET/CT is recommended for initial staging of non-small cell lung carcinoma in the absence of proven distant metastasis [15]. [^18^F]FLT, despite high diagnostic accuracy, is not routinely implemented in nuclear medicine departments due to challenges in production and to high costs. Data on SCLC date back to 2014; consequently, they do not reflect the eighth edition of the AJCC staging manual. The utility of diagnostic tests in this disease must be established through further investigations.

### Lung neuroendocrine tumour

For diagnostic purposes/characterisation, [^18^F]FDG PET/CT demonstrated a sensitivity of 0.71 (95% CI 0.66–0.76) [23]. On the other hand, somatostatin receptor targeted PET radiopharmaceuticals (e.g. DOTA-peptides) showed a sensitivity of 0.90 (95%CI 0.82–0.95) [23].

Summary of evidence & Comments Somatostatin receptor-targeted radiopharmaceutical PET/CT is the preferred imaging modality in the diagnostic setting of lung neuroendocrine tumour.

### Mesothelioma, thymoma, medullary thyroid cancer, neuroendocrine carcinoma

No meta-analyses have been selected for mesothelioma, thymoma, medullary thyroid carcinoma, and neuroendocrine carcinoma.

Summary of evidence & Comments provided in the Discussion section.

### Insulinoma

Glucagon-like peptide-1 receptor (GLP-1R) targeting PET/CT demonstrated a sensitivity and specificity of 0.79 (95% CI: 0.54–0.92) and 0.84 (95% CI: 0.20–0.99), respectively [24].

Summary of evidence & Comments PET/CT with GLP-1R-targeting radiopharmaceuticals is the preferred imaging modality in insulinomas. Somatostatin receptor-targeted radiopharmaceuticals can also be recommended [15].

### Differentiated thyroid cancer (DTC)

In the diagnostic setting of thyroid nodules with indeterminate cytology, [^18^F]FDG PET demonstrated a sensitivity and specificity of 0.95 (95%CI 0.82–0.99) and 0.58 (95%CI 0.51–0.66), respectively [25], The corresponding figures for PET/CT were 0.73 (95%CI 0.64–0.81) and 0.56 (95%CI 0.51–0.62), respectively [25].

In lymph-nodal staging of DTC, [^18^F]FDG PET/CT showed high specificity (0.94, 95%CI 0.92–0.95), while sensitivity was low (0.3, 95%CI 0.26–0.35) [26].

Summary of evidence & Comments [^18^F]FDG PET/CT showed low specificity in the diagnostic setting of primary lesions; low sensitivity was found for lymph-node metastases assessment. Other modalities than [^18^F]FDG PET/CT should be considered for diagnostic and staging purposes in DTC. [^124^I]I PET/CT is a sensitive modality to diagnose radioiodine avid DTC lesions, however, its clinical role has not been fully established yet [27].

### Breast cancer

For diagnostic purposes, the radiopharmaceutical and modality of choice is [^18^F]FDG PET/MRI, which on a per-lesion analysis demonstrated a sensitivity of 0.95 (95%CI 0.90–0.99) and a specificity of 0.91 (95%CI 0.84–0.96). On a per-patient analysis, sensitivity and specificity were 0.97 (95%CI 0.87-1.00) and 0.97 (95%CI 0.92–0.99), respectively [28]. For N staging, [^18^F]FDG PET/MRI is the modality with the highest diagnostic performance: sensitivity and specificity were 0.94 (95%CI 0.83–0.98) and 0.90 95% (CI 0.81–0.95), respectively [29]. The figures for [^18^F]FDG PET/CT were 0.64 (95%CI 0.59–0.69) and 0.93 (95%CI 0.90–0.95), respectively [30]. M staging with PET/MRI achieved sensitivity and specificity of 0.94 (95%CI 0.81–0.99) and 0.92 (95%CI 0.84–0.97), respectively, on a per-lesion analysis. On a per-patient analysis, the figures were 0.98 (95%CI 0.94-1.00) and 0.96 (95%CI 0.93–0.98), respectively [28]. On the other hand, the per-lesion sensitivity and specificity of [^18^F]FDG PET were found to be 0.69 and 0.98 and the per-patient ones were 0.78 and 0.79 [31].

Summary of evidence & Comments [^18^F]FDG PET/MRI resulted the most accurate imaging modality in breast cancer staging. However, it is not widely available. [^18^F]FDG PET/CT is recommended for staging IIB breast cancer and can be proposed for cT1cN1 or cT2cN0 tumours [15]. No meta-analyses were found on the diagnostic performance of PET imaging for different histological subtypes. 16α-[^18^F]Fluoro-17β-Estradiol ([^18^F]-FES) PET has been proposed to improve breast cancer diagnosis and staging, and to support therapy selection in metastatic disease patients [32]. Using IHC results as the reference standard, 16α-[^18^F]Fluoro-17β-Estradiol ([^18^F]-FES) PET sensitivity was 0.78 (95% CI 0.65–0.88) and specificity 0.98 (0.65–1.00) of non-breast lesions; while sensitivity was 0.86 (0.73–0.94) and specificity 0.76 (0.52–0.90) of breast lesions. In non-IHC tissue assays and all lesion sites, sensitivity resulted 0.81 (0.73–0.87) and specificity 0.86 (0.68–0.94) [33].

### Oesophageal cancer

Diagnostic performance of [^18^F]FDG PET/CT in N staging resulted in a per-patient sensitivity and specificity of 0.54 (95%CI 0.42–0.65) and 0.82 (95%CI 0.71–0.89), respectively; on a per-station basis, these figures were 0.63 (95%CI 0.38–0.83) and 0.96 (95%CI 0.94–0.98) [34]. Distant metastases detection by [^18^F]FDG PET achieved sensitivity and specificity of 0.71 (95%CI 0.62–0.79) and 0.93 (95%CI 0.89–0.97), respectively [35].

Summary of evidence & Comments [^18^F]FDG PET/CT is recommended for disease extension evaluation before radiochemotherapy or before surgery [15].

### Gastric cancer

The comparison between [^18^F]FDG and FAPI imaging (PET/CT and PET/MRI) revealed that FAPI PET had a detection rate of 1 in primary lesion identification vs. 0.84 of [18F]FDG PET. As for lymph-nodal metastases detection, FAPI showed a 0.82 detection rate vs. 0.67 of [^18^F]FDG. In peritoneal metastatic disease spread evaluation, FAPI demonstrated a 1.0 detection rate vs. 0.45 of [^18^F]FDG [36].

Summary of evidence & Comments Fibroblast activation protein inhibitors (FAPI) radiopharmaceuticals demonstrated high diagnostic accuracy, although they are still under investigation in clinical trials and have not yet received marketing authorization from any pharmaceutical regulatory authority worldwide. [^18^F]FDG PET/CT can be proposed to exclude regional lymph-node and distant metastases in patients affected by potentially resectable gastric cancer [15].

### Colorectal cancer

In the diagnostic setting of the primary lesion evaluation, [^18^F]FDG PET/MRI demonstrated a sensitivity and specificity of 0.95 (95%CI 0.31–1.00) and 0.79 95% (CI 0.52–0.93), respectively [37]. In the assessment of incidental findings, [^18^F]FDG PET/CT has been reported to have a sensitivity of 0.85 (95%CI 0.78–0.90) and a specificity of 0.87 (95%CI 0.77–0.94) [38]. In lymph-nodal staging, [^18^F]FDG PET/MRI showed 0.81 (95%CI 0.65–0.91) and 0.88 (95%CI 0.76–0.94) sensitivity and specificity, respectively [37]. In the same setting, [^18^F]FDG PET/CT was found to have a sensitivity of 0.66 (95%CI 0.63–0.69) and a specificity of 0.76 (95%CI 0.73–0.78) [39]. [^18^F]FDG PET/MRI demonstrated a sensitivity and specificity of 0.97 (95%CI 0.86–0.99) and 0.93 (95%CI 0.85–0.97), respectively, in distant metastases evaluation [37]. When focusing on liver metastases assessment, [^18^F]FDG PET or PET/CT showed a sensitivity and specificity of 0.93 (95%CI 0.88–0.96) and 0.93 (95%CI 0.84–0.98), respectively [40].

Summary of evidence & Comments [^18^F]FDG PET/MRI resulted more accurate than [^18^F]FDG PET/CT in colorectal cancer staging. However, PET/MRI is not widely available. [^18^F]FDG PET/CT is recommended in the pre-therapeutic setting in the presence of suspected metastases on other imaging tests. [^18^F]FDG PET/CT is recommended in patients with known and resectable metastases in order to exclude other metastatic sites. No meta-analyses were found on the diagnostic performance of PET imaging for different histological subtypes.

### Anal cancer

For primary lesion assessment, [^18^F]FDG PET or PET/CT had a sensitivity of 0.99 (95%CI 0.96-1.00) [41]. In nodal staging, the sensitivity and specificity of [^18^F]FDG PET/CT were found to be 0.56 (95%CI 0.45–0.67) and 0.90 (95%CI 0.86–0.93), respectively [42].

Summary of evidence & Comments [^18^F]FDG PET/CT is recommended for the purpose of disease staging; it is a specific modality for locoregional lymph node metastasis detection; however, sensitivity represents a concern.

### Pancreatic cancer

In primary disease evaluation, [^18^F]FDG PET/CT demonstrated 0.9 (95%CI 0.85–0.94) sensitivity and 0.8 (95%CI 0.73–0.86) specificity [43]. To identify lymph node metastases, [^18^F]FDG PET had a sensitivity of 0.64 (95%CI 0.50–0.76) and a specificity of 0.81 (95%CI 0.25–0.85) [44]. In liver metastases assessment, [^18^F]FDG PET/CT and PET/CT resulted in a sensitivity a specificity of 0.67 (95%CI 0.52–0.79) and 0.96 (95%CI 0.89–0.98), respectively [44].

Summary of evidence & Comments [^18^F]FDG PET/CT is a moderately accurate modality in the staging setting; [^18^F]FDG PET/CT and endoscopic ultrasound (EUS) could play complementary roles in pancreatic carcinoma assessment [43].

### Hepatic cell carcinoma

For metastatic spread assessment, [^18^F]FDG PET and PET/CT demonstrated a sensitivity and specificity of 0.76 (95%CI 0.68–0.83) and 0.98 (95%CI 0.92–0.99), respectively [45].

Summary of evidence & Comments [^18^F]FDG PET/CT and^11^C or^18^F -labelled choline PET/CT might be considered in selected cases [15, 45] PSMA PET/CT or PET/MR demonstrated a high detection rate [46]. In the meta-analysis published by Rizzo et al. [47], PSMA PET was compared to conventional imaging modalities that included contrast enhanced CT and/or MRI and/or FDG PET/CT. The pooled detection rate was 85.1% (95% CI: 77.9–90.7). Contrast-enhanced CT and MRI remain the gold-standard imaging methods for evaluating HCC, however, PSMA-targeting PET could be a complementary examination when conventional imaging is doubtful.

### Gallbladder cancer and cholangiocarcinoma

In gallbladder cancer, [^18^F]FDG PET and PET/CT had 0.87 (95%CI 0.82–0.92) sensitivity and 0.78 (95%CI 0.68–0.86) specificity for primary lesion evaluation [48].

In cholangiocarcinoma primary lesion staging, [^18^F]FDG PET and PET/CT showed sensitivity and specificity of 0.81 (95%CI 0.78–0.83) and 0.82 (95%CI 0.75–0.87), respectively [49]. For nodal staging, the same modality demonstrated 0.52 (95%CI 0.44–0.60) and 0.91 (95%CI 0.87–0.95) sensitivity and specificity, respectively [50]. In cholangiocarcinoma M staging, [^18^F]FDG PET/CT was found to have 0.56 (95%CI 0.42–0.69) and 0.95 (95%CI 0.91–0.97) sensitivity and specificity, respectively [51].

Summary of evidence & Comments [^18^F]FDG PET/CT can be proposed for initial staging to identify regional lymph-nodal and distant metastases in patients amenable to surgical resection.

### Gastro-entero-pancreatic neuroendocrine tumours (GEP NET)

From the comparison between [^18^F]FDG and DOTA-peptides imaging (PET and PET/CT), [^18^F]FDG, when used for primary diagnosis, demonstrated a sensitivity of 0.7 (95%CI 0.41–0.89) and a specificity of 0.97 (95%CI 0.70-1.00). In contrast, DOTA-peptides targeting somatostatin receptors for PET and PET/CT imaging achieved, on a per-patient basis, a sensitivity of 0.92 (95%CI 0.89–0.95) and a specificity of 0.91 (95%CI 0.83–0.95), respectively [52]. Lesion characterisation by [^18^F]DOPA PET and PET/CT had a sensitivity of 0.83 (95%CI 0.70–0.92) and 0.95 (95%CI 0.89–0.99) on a per-patient and per-lesion analysis, respectively [53].

Summary of evidence & Comments Somatostatin receptor-targeted radiopharmaceutical PET/CT is the preferred imaging modality in the diagnostic setting in GEP NETs. In case of negative DOTA-peptides imaging, [^18^F]FDG is recommended. [^18^F]FDG provides complementary information and thus can be performed in GEP NET of any grade if a positive result is expected to change patient management [54].

### Pheochromocytoma and paraganglioma

DOTA-peptides targeting somatostatin receptors PET had a sensitivity of 0.93 (95%CI 0.91–0.95) [55] for the identification of pheochromocytoma and paraganglioma. In lymph-node and distant metastases staging of disease with germline mutations, [^18^F]FDG PET/CT demonstrated 0.83 (95%CI 0.68–0.92) and 0.74 (95%CI 0.14–0.98) sensitivity and specificity, respectively [56].

Summary of evidence & Comments Somatostatin receptor-targeted radiopharmaceutical PET/CT is recommended for lesion detection. [^18^]DOPA PET/CT can also be considered. [^18^F]FDG PET/CT can be proposed in specific cases (e.g. patients with succinate dehydrogenase subunit B (SDHB) mutation) [15].

### Renal cell carcinoma

For primary lesion diagnosis, [^18^F]FDG PET had a sensitivity and specificity of 0.83 (95%CI 0.64–0.93) and 0.86 (95%CI 0.75–0.92), respectively. The figures for [^18^F]FDG PET/CT were 0.89 (95%CI 0.72–0.96) and 0.88 (95%CI 0.76–0.95), respectively [57]. As for lymph node and distant metastases staging, [^18^F]FDG PET demonstrated a sensitivity and specificity of 0.79 (95%CI 0.71–0.86) and 0.90 (95%CI 0.82–0.95) on a per-patient basis, and of 0.84 (95%CI 0.75–0.91) and 0.91 (95%CI 0.72–0.99) on a per-lesion basis [57].

Summary of evidence & Comments[^18^F]FDG PET/CT has limited utility for renal cell carcinoma, due to its renal excretion, particularly in clear cell variant. To characterise renal lesions, carbonic anhydrase IX-targeted radiopharmaceuticals can be proposed [58]. Two phase III trials have evaluated the role of girentuximab PET/CT in patients with renal masses. In particular, ^124^I-labeled girentuximab detected clear cell RCC with sensitivity of 86.2% (95% CI 75.3–97.1%) and 85.9% (95% CI 69.4%–9.9%), respectively ((REDECT) trial [59]). In the ZIRCON trial, the sensitivity and specificity of ^89^Zr-DFO-girentuximab PET/CT in detecting ccRCC in patients with indeterminate renal masses, using histology as the standard of reference, were 85.5% and 87%, respectively [60]. No meta-analyses were found on the diagnostic performance of PET imaging for different histological subtypes.

### Bladder cancer

In bladder cancer primary tumour detection, [^18^F]FDG PET and PET/CT demonstrated a sensitivity and specificity of 0.80 (95%CI 0.71–0.87) and 0.84 (CI 0.69–0.93), respectively, on a per-lesion basis [61]. In included studies, to better interpret bladder lesions pelvic delayed scans, furosemide administration, or a catheter were applied [61]. In lymph-node staging, [^18^F]FDG PET/CT showed a sensitivity of 0.56 (95%CI 0.47–0.65) and a specificity of 0.95 (95%CI 0.92–0.98) [62].

Summary of evidence & Comments [^18^F]FDG PET/CT cannot be recommended for diagnostic and staging purposes in bladder cancer.

### Ovarian cancer

[^18^F]FDG PET has proven to be useful in ovarian cancer. In primary lesion characterisation, sensitivity resulted 0.94 95% (CI 0.87–0.97) and specificity 0.86 (95%CI 0.79–0.91) [63]. As for nodal metastases detection, PET and PET/CT demonstrated a sensitivity and specificity of 0.73 (95%CI 0.68–0.78) and 0.97 (95%CI 0.96–0.98), respectively [64]. Metastatic disease staging by PET and PET/CT yielded, on per-site analysis, a sensitivity of 0.72 (95%CI 0.61–0.81) and a specificity of 0.93 (95%CI 0.85–0.97) [65].

Summary of evidence & Comments [^18^F]FDG PET/CT can be proposed for the loco-regional or remote extension assessment of advanced ovarian cancer [15]. No meta-analyses were found on the diagnostic performance of PET imaging for different histological subtypes.

### Endometrial cancer

In the diagnostic setting of endometrial cancer, [^18^F]FDG PET and PET/CT resulted in a sensitivity and a specificity of 0.82 (95%CI 0.78–0.85) and 0.90 (95%CI 0.79–0.96), respectively [66]. From the per-patient analysis, nodal staging by PET/CT achieved 0.67 (CI 0.61–0.73) sensitivity and 0.91 (95%CI 0.87–0.94) specificity [67]. Distant metastases detection by PET and PET/CT resulted in 0.96 (95%CI 0.85–0.99) sensitivity and 0.96 (95%CI 0.93–0.97) specificity [66].

Summary of evidence & Comments [^18^F]FDG PET/CT can be proposed for staging in the context of a high risk of metastatic disease (≥ Stage FIGO II) [15].

### Cervical cancer

The review criteria allowed the selection of a study focused on nodal staging. The [^18^F]FDG PET/CT per-lesion analysis demonstrated a sensitivity of 0.55 (95%CI 0.44–0.65), while specificity was 0.98 (95%CI 0.96–0.99). On a per-patient analysis, the same figures were 0.76 (95%CI 0.60–0.87) and 0.94 (95%CI 0.91–0.96), respectively [68].

Summary of evidence & Comments [^18^F]FDG PET/CT is highly recommended for cervical cancer staging [15].

### Vulvar cancer

Lymph-nodal staging in vulvar cancer by [^18^F]FDG PET and PET/CT yielded sensitivity and specificity of 0.7 (95%CI 0.44–0.95) and 0.9 (95%CI 0.76-1.00) on a per-patient analysis, of 0.76 (95%CI 0.57–0.94) and 0.88 (95%CI 0.82–0.94) on a per-region analysis, and of 0.62 (95%CI 0.41–0.80) and 0.91 (95%CI 0.80–0.97) on a per-lesion analysis, respectively [69].

Summary of evidence & Comments [^18^F]FDG PET/CT can be considered for vulval cancer lymph-node staging in selected cases.

### Prostate cancer

For primary tumour detection, 3 meta-analyses assessing the diagnostic performance of different radiopharmaceuticals were selected. [^68^Ga]Ga-PSMA-11 PET/CT demonstrated sensitivity and specificity of 0.97 (95%CI 0.90–0.99) and 0.66 (95%CI 0.52–0.78), respectively [70]. [^18^F]PSMA-1007 PET/CT showed a sensitivity of 0.96 (95%CI 0.94–0.98), while specificity was not assessed [71]. Using Fluciclovine, PET/CT resulted in a 0.85 (95%CI 0.73–0.92) sensitivity and 0.77 (95%CI 0.60–0.88) specificity [72].

In nodal staging, [^68^Ga]Ga-PSMA-11 showed 0.61 (95%CI 0.39–0.79) sensitivity and 0.96 (95%CI 0.92–0.98) specificity on a per-patient basis; the figures were 0.74 (CI 0.50–0.89) and 0.99 (95%CI 0.98-1.00) on a per-lesion basis [73]. PET/MRI using PSMA-targeted compounds showed a sensitivity of 0.67 (95%CI 0.50–0.81) and specificity of 0.93 (95%CI 0.88–0.97) on a per-patient basis, while the same figures were 0.64 (95%CI 0.44–0.81) and 0.97 (95%CI 0.91–0.99) on a per-lesion analysis [74]. A small size of the metastatic deposits within the lymph nodes led to a lower sensitivity of the method.

Bone metastases detection by [^68^Ga]Ga-PSMA-11 PET/CT demonstrated a sensitivity and specificity of 0.97 (95%CI 0.92–0.99) and 1.00 (95%CI 0.98-1.00), respectively [75]. Choline radiopharmaceuticals (both ^11^C- and ^18^F-labelled) showed sensitivity and specificity of 0.87 (95%CI 0.80–0.92) and 0.99 (95%CI 0.96-1), respectively [76]. Finally, the bone-seeking compound [^18^F]NaF showed a sensitivity and specificity of 0.96 (95%CI 0.87–0.99) and 0.97 (95%CI 0.90–0.99), respectively [76].

Summary of evidence & Comments PSMA-targeted PET/CT or PET/MRI can be recommended for the staging of high-risk patients (Gleason score > 7, PSA > 20 ng/mL, clinical stage T2c – 3a) eligible to treatments with curative intent. Fluciclovine, [^11^C] and [^18^F]-labelled choline PET/CT or PET/MRI can be proposed in the same settings [15].

### Testicular cancer

In testicular cancer diagnosis, [^18^F]FDG PET/CT resulted in a sensitivity and specificity of 0.75 (95% CI 0.70–0.80) and 0.87 (95% CI 0.84–0.89), respectively [77].

Summary of evidence& Comments [^18^F]FDG PET/CT can be proposed for initial staging. No meta-analyses were found on the diagnostic performance of PET imaging focused on the diagnosis and staging of seminoma, non-seminoma or teratoma.

### Penile cancer

The review criteria allowed us to identify a study focused on lymph nodal staging, which demonstrated that [^18^F]FDG PET/CT had a sensitivity of 0.81 (95% CI 0.70–0.89) and a specificity of 0.92 (95% CI 0.87–0.96) [78].

Summary of evidence & Comments [^18^F]FDG PET/CT can be proposed for initial lymph-nodal staging.

### Melanoma

On a per-patient basis, [^18^F]FDG PET/CT resulted in a sensitivity and specificity of 0.89 (95%CI 0.65–0.97) and 0.88 (95%CI 0.77–0.94), respectively, in lymph-nodal metastases detection [79].

Summary of evidence & Comments [^18^F]FDG PET/CT is recommended for staging cutaneous melanoma with macroscopic lymph-node metastasis or at high risk for distant disease locations (stage IIIB-C). In cutaneous melanoma, before surgery, [^18^F]FDG PET/CT is recommended for staging in patients with resectable macroscopic lymph-node location (stage IIIB-C) and cutaneous stage IV disease with a presumed isolated metastatic lesion [15].

### Merkel cell carcinoma

Nodal staging by [^18^F]FDG PET/CT achieved a sensitivity and a specificity of 0.91 (95%CI 0.85–0.95) and 0.93 95% (CI 0.86–0.97), respectively [80].

Summary of evidence & Comments [^18^F]FDG PET/CT can be proposed for initial lymph-nodal staging. In case of nodal or distant metastases from unknown primary Merkel Cell Carcinoma and negative [^18^F]FDG PET/CT, DOTA-peptide imaging can be considered.

### Ewing sarcoma

For primary lesion assessment, [^18^F]FDG PET or PET/CT showed 0.97 (95%CI 0.92–0.99) sensitivity and 0.68 (CI 0.44–0.86) specificity [81]. In the same study, the authors found that [^18^F]FDG PET had a sensitivity and specificity of 0.79 (95%CI 0.59–0.91) and 0.98 (95%CI 0.94–0.99), respectively, for lymph-nodal staging [81]. In the detection of lung metastases, [^18^F]FDG PET or PET/CT demonstrated a sensitivity and specificity of 0.76 (95%CI 0.61–0.87) and 0.92 (95%CI 0.86–0.96), respectively [81], with CT as a superior modality for lung metastasis detection. For bone metastases detection, [^18^F]FDG PET or PET/CT showed a sensitivity and specificity of 0.91 (95%CI 0.80–0.97) and 0.98 (95%CI 0.94–0.99), respectively [82].

Summary of evidence & Comments [^18^F]FDG PET/CT is recommended for the purpose of staging Ewing Sarcoma. Bone marrow biopsy and/or aspirate can be omitted in patients with otherwise localized disease after initial staging studies [79].

### Chondrosarcoma

On a per-patient analysis, [^18^F]FDG PET/CT demonstrated a sensitivity and specificity of 0.94 (95%CI 0.86–0.97) and 0.89 (95%CI 0.82–0.93), respectively, in primary chondrosarcoma assessment [83].

Summary of evidence & Comments [^18^F]FDG PET/CT can be proposed for initial staging.

## Discussion

The present umbrella review indicates that, in modern PET/CT imaging of solid tumours, a wide array of radiopharmaceuticals is available, as the choice of the correct option for the clinical scenario at hand is ever more complex. This work aims to guide the correct diagnostic workup in adult extracranial solid tumours. In general, radiopharmaceuticals can be divided into two broad categories: tracers of metabolism and tracers of specific tumour-related processes. The first group includes analogues of glucose, amino acids, and membrane fatty compounds; their advantages lie in targeting the key characteristic of malignant neoplasms, i.e. accelerated and uncontrolled growth. On the other hand, they might show suboptimal diagnostic efficiency in slow-growing entities and may lack in specificity. The study confirmed the central role of [^18^F]FDG PET in this class of radiopharmaceuticals and overall in evaluating solid malignancies. Other broadly used tracers belonging to this group are DOPA and Choline. The second group includes molecules targeting specific functional aspects of the cells from which the tumour originated, such as the expression of somatostatin receptors. Their main advantages include high specificity for the target tissue and the possibility of theranostic applications; their disadvantage is that they might lose sensitivity as the target tumour becomes less differentiated. This group features widely used radiopharmaceuticals, such as DOTA-peptides and PSMA tracers, along with other tracers, some of which are currently under testing such as [^18^F]fluoroestradiol for oestrogen receptors in breast cancer, [^124^I]Iodine for differentiated thyroid cancer, and [^18^F]metafluorobenzylguanidine for neuroblastoma. Other radiopharmaceuticals, such as FAPI and natrium fluoride, belong to a third, less represented but promising category: tumour microenvironment tracers.

Overall, the current evidence indicates [^18^F]FDG as the main player in molecular imaging, with DOTA-peptides, PSMA tracers, and a handful of other compounds complementing the glucose analogue and guiding theranostic applications. PSMA tracers are used in staging and re-staging prostate cancer, while somatostatin-targeting peptides (e.g. [^68^Ga]Ga-DOTA-TOC and -TATE) or [^18^F]DOPA are widely employed in neuroendocrine tumours [84–86]. FAPI has emerged in gastric disease assessment. These findings can inform guideline updates, to offer patients the best available diagnostic options.

The present review does not stratify the diagnostic performance results according to disease stage and grading, which might be relevant is some conditions. When approaching a particular cancer type guideline, specific considerations about the diagnostic accuracy and clinical impact should be made. Still, we aimed to identify the most robust evidence focusing on the biological and metabolic characteristics according to histology of the diseases. We also reported, when available, diagnostic performance results in primary, lymph-nodal, and distant metastases detection.

Besides providing indications on the current PET tracers, the present review highlighted that high-level evidence on PET/CT diagnostic performance is lacking in several oncological diseases and indications, as detailed in Table 1. Research efforts should be focused to bridge these gaps. Indeed, eligible meta-analyses were not found for mesothelioma, thymoma, medullary thyroid cancer, neuroendocrine carcinoma, soft tissue sarcomas, gastrointestinal stromal tumours (GIST) and other (ultra)rare cancers. This lack of evidence might be explained based on the rarity and/or heterogeneity of the diseases and their specific characteristics. Mesothelioma is an aggressive malignancy with high [^18^F]FDG uptake; a possible caveat is, however, represented by talc administration to treat the frequently associated malignant pleural effusion, causing a high-grade and persistent inflammation, which might hamper follow-up with this imaging modality. Thymoma is an exceedingly rare disease [87], which hinders the gathering of a significant volume of scientific evidence; however, being an epithelial-derived disease, it is likely that [^18^F]FDG is the tracer of choice. Medullary thyroid cancer is also rare, yet abundant studies suggest that [^18^F]DOPA is the best-performing tracer in staging nodal and distant localisations and in detecting tumour recurrences. Neuroendocrine carcinoma, as well as poorly differentiated neuroendocrine neoplasms, show increased uptake of [^18^F]FDG. [^18^F]FDG PET/CT, when positive, can identify lesions with a more aggressive behaviour and worse prognosis [88]. In soft tissues sarcomas with seventy subtypes, sufficient evidence on the role of PET imaging is lacking. Similarly, sub-analyses in different histological types and in different receptor expression profile cancers are necessary to provide personalised guidance of patient management.

PSMA is known to be overexpressed in cancers other than prostate cancer; indeed, its presence has been demonstrated in the neo-vasculature of different cancer entities, such as clear-cell renal cancer and hepatic cell carcinoma. Moreover, it is known that PSMA is overexpressed on the membrane of salivary gland cancer cells [89]. However, data on these topics remain limited and further multicentric studies are necessary.

During the last few years, FAPI PET radiopharmaceuticals have gained increasing interest due to their favourable biodistribution and superior performance compared to [^18^F]FDG in the identification of many neoplastic entities, including liver metastases, peritoneal carcinomatosis, and primary gastrointestinal, lung, bladder, and ovarian tumours [90]. However, few insights on the role of FAPI PET/CT imaging are currently available in diseases that are known to overexpress FAP on the malignant cell membrane itself [91], like certain sarcomas, or on the cell membrane of activated fibroblasts within the microenvironment of salivary gland cancer, cholangiocarcinoma, breast cancer, and others.

[^18^F]DOPA, a radiolabelled dopamine precursor that enters cells through L-amino acid transporters, represents a valid alternative to radiolabelled somatostatin analogues for imaging NETs. Moreover, DOPA represents a versatile tracer, which can be used in brain neoplasms and even in non-oncological indications, such as basal ganglia evaluation in suspected Parkinson’s disease. However, due to its high costs, low availability, and technically difficult production [92], PET with [^18^F]DOPA is currently restricted in the oncological field to the evaluation of tumours with low or variable SSTR expression, including primitive neuroectodermal tumours (PNETs), medullary thyroid cancer, jejune-ileal NET, pheochromocytoma, neuroblastoma and paraganglioma [93].

In recent years, radiopharmaceutical research focused on theranostic compounds. The success of radiolabelled somatostatin analogues for imaging and radioligand therapy in tumours expressing somatostatin receptors paved the way for developing a broader panel of radiolabelled peptides targeting different tumours [94]. Theranostics implies that the same, or a similar molecule can be labelled with an isotope suitable for imaging, but also with another isotope capable of releasing a high amount of energy, suitable for treatment purposes (radioligand therapy, RLT). Molecules targeting C-X-C chemokine receptor type 4 (CXCR-4) [95] for haematological malignancies, neurotensin receptor 1 (NTR1) for pancreatic adenocarcinoma [96], cholecystokinin B receptor (CCK2-R) [97], the gastrin-releasing peptide receptor (GRP-R) [98], and integrin receptors [99] for multiple cancers are expected to represent valuable theranostic approaches in the management of these malignancies. Trends in investments by pharmaceutical companies confirm a major interest in the field [100]. In this sense, the exact definition of the most appropriate diagnostic tracer for every theranostic compound is of pivotal importance. However, it must be considered that the mere uptake positivity on static images might not necessarily indicate success in the subsequent therapy, as demonstrated by the recent FAPI theranostic trial experiences, showing variable results [101–103]. Multiple time points of evaluation might be needed to predict the dynamic behaviour of the theranostic radiopharmaceutical in each setting.

Some meta-analyses, which have been included according to selection criteria, report data obtained on PET only. This may result in bias towards older papers with inferior scanner technology, which may lead to an underestimate of PET’s performance as compared to current PET/CT systems. Nowadays, PET alone is an obsolete technology and should not be recommended.

This study has some limitations. First, no original research articles were included since the aim was to provide the highest level of evidence, a goal achieved through the rigorous evaluation conducted by five independent reviewers. Notably, the use of very stringent criteria for assessing evidence resulted in high agreement in scores, maximising an objective evaluation of the papers. The few minimal differences in ratings were easily resolved through discussion. Secondly, as this umbrella review concerned the application of PET imaging specifically, no comparisons with other imaging modalities (CT, MRI, scintigraphy) were undertaken. Thirdly, we excluded haematological malignancies. Moreover, even we focused only on solid tumours, unknown primary tumours were not included in the analysis; however, previous meta-analyses demonstrated [^18^F]FDG usefulness for both primary tumour detection and staging [104, 105]. Finally, some of the radiopharmaceuticals that passed the selection criteria of this umbrella review, such as [^18^F]FLT or [^18^F]NaF, are currently scarcely used, either because of reduced availability or because of the rise of better alternatives.

In conclusion, as our understanding of oncological diseases continues to deepen, accompanied by advancements in biological, molecular, and genetic research, the array of PET radiopharmaceuticals at our disposal expands. The comprehensive evidence synthesised in the present umbrella review serves as a guiding compass for clinicians and imagers, aiding them in navigating the increasingly intricate seascape of PET examinations. In an era defined by personalised medicine and theranostics, the insights provided here offer clarity amidst the complexity, facilitating informed decision-making for improved patient care.

## Electronic supplementary material

Below is the link to the electronic supplementary material.

Supplementary Material 1

## Data Availability

All data generated or analysed during this study are included in this published article and its supplementary information files.
