# Supplementary material for: Personalised PET imaging in oncology: an umbrella review of meta-analyses to guide the appropriate radiopharmaceutical choice and indication
Source: Eur J Nucl Med Mol Imaging. 2024 Sep 11;52(1):208–24. doi: 10.1007/s00259-024-06882-9 (PMC11599298; doi:10.1007/s00259-024-06882-9)
Supplement: Supplementary file 1 — Supplementary Material 1 [file 259_2024_6882_MOESM1_ESM.docx]

**Supplementary material**

**Personalised PET Imaging in Oncology: An Umbrella Review of Meta-Analyses to Guide the Appropriate Radiopharmaceutical Choice and Indication**

**Margarita Kirienko^1^*, Fabrizia Gelardi^2^*, Francesco Fiz^3,4^, Matteo Bauckneht^5,6^, Gaia Ninatti^7,8§^, Cristiano Pini^7,8^, Alberto Briganti^2,9^, Massimo Falconi^2,10^, Wim J G Oyen^11,12,13^, Winette T A van der Graaf^14,15^, Martina Sollini^2,7^**

*Contributed equally

§Corresponding author

1 – Nuclear Medicine, Fondazione IRCCS Istituto Nazionale dei Tumori di Milano, Milan, Italy

2 – Vita-Salute San Raffaele University, Via Olgettina 58, Milan, 20132, Italy

3 – Department of Nuclear Medicine, E.O. “Ospedali Galliera”, Genoa, Italy

4 – Department of Nuclear Medicine and Clinical Molecular Imaging, University Hospital, Tübingen, Germany

5 - Department of Health Science (DISSAL), University of Genoa, Genoa, Italy

6 - Nuclear Medicine, IRCCS Ospedale Policlinico San Martino, Genoa, Italy

7 –Department of Nuclear Medicine, IRCCS Ospedale San Raffaele, Milan, 20132, Italy.

8 - School of Medicine and Surgery, University of Milano-Bicocca, Monza, Italy

9 - Division of Oncology/Unit of Urology, URI, IRCCS Ospedale San Raffaele, Milan, Italy

10 - Pancreatic and Transplant Surgery Unit, Vita-Salute University, San Raffaele Hospital, Milan, Italy

11 - Department of Radiology and Nuclear Medicine, Rijnstate Hospital, Arnhem, The Netherlands.

12 - Department of Biomedical Sciences, Humanitas University, Milan, Italy.

13 - Department of Nuclear Medicine, Humanitas Clinical and Research Center, Milan, Italy.

14 - Department of Medical Oncology, Netherlands Cancer Institute - Antoni van Leeuwenhoek, Amsterdam, The Netherlands

15 - Department of Medical Oncology, Erasmus MC Cancer Institute, Erasmus University Medical Center, Rotterdam, The Netherlands

**Supplementary Table 1: Search algorithm for each disease and number of results**

HNSCC (oral cavity, oropharynx, nasal cavity and paranasal sinuses, nasopharynx, larynx, hypopharynx)

01/04/2023

“mouth” AND (“FDG” OR “PSMA” OR “FAPI” OR “DOPA” OR “DOTATOC”) AND “PET”

“lip” AND (“FDG” OR “PSMA” OR “FAPI” OR “DOPA” OR “DOTATOC”) AND “PET”

“tongue” AND (“FDG” OR “PSMA” OR “FAPI” OR “DOPA” OR “DOTATOC”) AND “PET”

“oral” AND (“FDG” OR “PSMA” OR “FAPI” OR “DOPA” OR “DOTATOC”) AND “PET”

“oropharynx" AND (“FDG” OR “PSMA” OR “FAPI” OR “DOPA” OR “DOTATOC”) AND "PET”

“oropharyngeal" AND (“FDG” OR “PSMA” OR “FAPI” OR “DOPA” OR “DOTATOC”) AND "PET”

“nasal" AND (“FDG” OR “PSMA” OR “FAPI” OR “DOPA” OR “DOTATOC”) AND "PET”

“paranasal" AND (“FDG” OR “PSMA” OR “FAPI” OR “DOPA” OR “DOTATOC”) AND "PET”

“sinus” AND (“FDG” OR “PSMA” OR “FAPI” OR “DOPA” OR “DOTATOC”) AND "PET”

"nasopharynx" AND (“FDG” OR “PSMA” OR “FAPI” OR “DOPA” OR “DOTATOC”) AND "PET"

"nasopharyngeal" AND (“FDG” OR “PSMA” OR “FAPI” OR “DOPA” OR “DOTATOC”) AND "PET"

"rhinopharynx" AND (“FDG” OR “PSMA” OR “FAPI” OR “DOPA” OR “DOTATOC”) AND "PET"

"rhinopharyngeal" AND (“FDG” OR “PSMA” OR “FAPI” OR “DOPA” OR “DOTATOC”) AND "PET"

“larynx” AND (“FDG” OR “PSMA” OR “FAPI” OR “DOPA” OR “DOTATOC”) AND "PET”

“laryngeal” AND (“FDG” OR “PSMA” OR “FAPI” OR “DOPA” OR “DOTATOC”)AND "PET”

“hypopharynx” AND (“FDG” OR “PSMA” OR “FAPI” OR “DOPA” OR “DOTATOC”) AND "PET”

“hypopharyngeal” AND (“FDG” OR “PSMA” OR “FAPI” OR “DOPA” OR “DOTATOC”) AND "PET”

“HNSCC” AND (“FDG” OR “PSMA” OR “FAPI” OR “DOPA” OR “DOTATOC”) AND "PET”

“head and neck” AND (“FDG” OR “PSMA” OR “FAPI” OR “DOPA” OR “DOTATOC”) AND "PET”

46 results

Salivary gland cancer

01/04/2023

“salivary” AND (“FDG” OR “PSMA” OR “FAPI” OR “DOPA” OR “DOTATOC”) AND "PET”

0 results

Anaplastic thyroid cancer

01/04/2023

“Anaplastic thyroid” AND (“FDG” OR “PSMA” OR “FAPI” OR “DOPA” OR “DOTATOC”) AND "PET”

0 results

Medullary thyroid cancer

01/04/2023

“Medullary thyroid” AND (“FDG” OR “PSMA” OR “FAPI” OR “DOPA” OR “DOTATOC”) AND "PET”

3 results

Lacrimal gland cancer

01/04/2023

“Lacrimal” AND (“FDG” OR “PSMA” OR “FAPI” OR “DOPA” OR “DOTATOC”) AND "PET”

0 results

Lung NENs (typical carcinoid, atypical carcinoid, LCNEC and SCLC)

01/04/2023

“carcinoid” AND (“FDG” OR “PSMA” OR “FAPI” OR “DOPA” OR “DOTATOC”) AND "PET”

“lung” AND “neuroendocrine” AND (“FDG” OR “PSMA” OR “FAPI” OR “DOPA” OR “DOTATOC”) AND "PET”

“LCNEC” AND (“FDG” OR “PSMA” OR “FAPI” OR “DOPA” OR “DOTATOC”) AND "PET”

“SCLC” AND (“FDG” OR “PSMA” OR “FAPI” OR “DOPA” OR “DOTATOC”) AND "PET”

3 results

Mesothelioma

01/04/2023

“mesothelioma” AND (“FDG” OR “PSMA” OR “FAPI” OR “DOPA” OR “DOTATOC”) AND "PET”

2 results

Thymoma

01/04/2023

“thymoma” AND (“FDG” OR “PSMA” OR “FAPI” OR “DOPA” OR “DOTATOC”) AND "PET”

“thymus” AND (“FDG” OR “PSMA” OR “FAPI” OR “DOPA” OR “DOTATOC”) AND "PET”

“thymic” AND (“FDG” OR “PSMA” OR “FAPI” OR “DOPA” OR “DOTATOC”) AND "PET”

4 results

Esophageal cancer

01/04/2023

“esophageal” AND (“FDG” OR “PSMA” OR “FAPI” OR “DOPA” OR “DOTATOC”) AND "PET”

“esophagus” AND (“FDG” OR “PSMA” OR “FAPI” OR “DOPA” OR “DOTATOC”) AND "PET”

17 results

Gastric cancer

01/04/2023

“gastric” AND (“FDG” OR “PSMA” OR “FAPI” OR “DOPA” OR “DOTATOC”) AND "PET”

“stomach” AND (“FDG” OR “PSMA” OR “FAPI” OR “DOPA” OR “DOTATOC”) AND "PET”

10 results

Pancreatic cancer

01/04/2023

“pancreatic” AND (“FDG” OR “PSMA” OR “FAPI” OR “DOPA” OR “DOTATOC”) AND "PET”

“pancreas” AND (“FDG” OR “PSMA” OR “FAPI” OR “DOPA” OR “DOTATOC”) AND "PET”

17 results

Hepatocellular carcinoma

01/04/2023

“HCC” AND (“FDG” OR “PSMA” OR “FAPI” OR “DOPA” OR “DOTATOC”) AND "PET”

“liver cancer” AND (“FDG” OR “PSMA” OR “FAPI” OR “DOPA” OR “DOTATOC”) AND "PET”

4 results

GEP NETs

01/04/2023

“NET” AND (“FDG” OR “PSMA” OR “FAPI” OR “DOPA” OR “DOTATOC”) AND "PET”

“neuroendocrine” AND (“FDG” OR “PSMA” OR “FAPI” OR “DOPA” OR “DOTATOC”) AND "PET”

25 results

“insulinoma” AND (“FDG” OR “PSMA” OR “FAPI” OR “DOPA” OR “DOTATOC”) AND "PET”

25 results

Neuroendocrine carcinoma

“neuroendocrine carcinoma” AND (“FDG” OR “PSMA” OR “FAPI” OR “DOPA” OR “DOTATOC”) AND "PET”

“NEC” AND (“FDG” OR “PSMA” OR “FAPI” OR “DOPA” OR “DOTATOC”) AND "PET”

3 results

Small bowel cancer

01/04/2023

“small bowel” AND (“FDG” OR “PSMA” OR “FAPI” OR “DOPA” OR “DOTATOC”) AND "PET”

“small intestine” AND (“FDG” OR “PSMA” OR “FAPI” OR “DOPA” OR “DOTATOC”) AND "PET”

“small intestinal” AND (“FDG” OR “PSMA” OR “FAPI” OR “DOPA” OR “DOTATOC”) AND "PET”

0 results

Cholangiocarcinoma and gallbladder cancer

01/04/2023

“cholangiocarcinoma” AND (“FDG” OR “PSMA” OR “FAPI” OR “DOPA” OR “DOTATOC”) AND "PET”

“bile duct” AND (“FDG” OR “PSMA” OR “FAPI” OR “DOPA” OR “DOTATOC”) AND “PET”

“biliary” AND (“FDG” OR “PSMA” OR “FAPI” OR “DOPA” OR “DOTATOC”) AND “PET”

“gallbladder” AND (“FDG” OR “PSMA” OR “FAPI” OR “DOPA” OR “DOTATOC”) AND "PET”

6 results

Adrenal gland cancer

01/04/2023

“adrenal” AND (“FDG” OR “PSMA” OR “FAPI” OR “DOPA” OR “DOTATOC”) AND "PET”

8 results

Vaginal cancer

01/04/2023

“vagina” AND (“FDG” OR “PSMA” OR “FAPI” OR “DOPA” OR “DOTATOC”) AND "PET”

“vaginal” AND (“FDG” OR “PSMA” OR “FAPI” OR “DOPA” OR “DOTATOC”) AND "PET”

0 results

Vulvar cancer

01/04/2023

“vulva” AND (“FDG” OR “PSMA” OR “FAPI” OR “DOPA” OR “DOTATOC”) AND "PET”

“vulvar” AND (“FDG” OR “PSMA” OR “FAPI” OR “DOPA” OR “DOTATOC”) AND "PET”

1 result

Anal cancer

01/04/2023

“anus” AND (“FDG” OR “PSMA” OR “FAPI” OR “DOPA” OR “DOTATOC”) AND "PET”

“anal” AND (“FDG” OR “PSMA” OR “FAPI” OR “DOPA” OR “DOTATOC”) AND "PET”

“anorectum” AND (“FDG” OR “PSMA” OR “FAPI” OR “DOPA” OR “DOTATOC”) AND "PET”

3 results

Penile cancer

01/04/2023

“penis” AND (“FDG” OR “PSMA” OR “FAPI” OR “DOPA” OR “DOTATOC”) AND "PET”

“penile” AND (“FDG” OR “PSMA” OR “FAPI” OR “DOPA” OR “DOTATOC”) AND "PET”

2 results

Merkel cell carcinoma

01/04/2023

“Merkel” AND (“FDG” OR “PSMA” OR “FAPI” OR “DOPA” OR “DOTATOC”) AND "PET”

2 results

Sarcoma (bone and joint, soft tissue)

01/04/2023

“sarcoma” AND (“FDG” OR “PSMA” OR “FAPI” OR “DOPA” OR “DOTATOC”) AND "PET”

17 results

Differentiated thyroid cancer (papillary, follicular)

01/04/2023

“thyroid” AND (“FDG” OR “PSMA” OR “FAPI” OR “DOPA” OR “DOTATOC”) AND "PET”

28 results

NSCLC

01/04/2023

“lung” AND (“FDG” OR “PSMA” OR “FAPI” OR “DOPA” OR “DOTATOC”) AND "PET”

“NSCLC” AND (“FDG” OR “PSMA” OR “FAPI” OR “DOPA” OR “DOTATOC”) AND "PET”

73 results

Breast cancer

01/04/2023

“breast” AND (“FDG” OR “PSMA” OR “FAPI” OR “DOPA” OR “DOTATOC”) AND "PET”

32 results

Colo-rectal cancer

01/04/2023

“colon” AND (“FDG” OR “PSMA” OR “FAPI” OR “DOPA” OR “DOTATOC”) AND "PET”

“colorectal” AND (“FDG” OR “PSMA” OR “FAPI” OR “DOPA” OR “DOTATOC”) AND "PET”

“rectum” AND (“FDG” OR “PSMA” OR “FAPI” OR “DOPA” OR “DOTATOC”) AND "PET”

“rectal” AND (“FDG” OR “PSMA” OR “FAPI” OR “DOPA” OR “DOTATOC”) AND "PET”

35 results

Kidney cancer

01/04/2023

“kidney” AND (“FDG” OR “PSMA” OR “FAPI” OR “DOPA” OR “DOTATOC”) AND "PET”

“renal” AND (“FDG” OR “PSMA” OR “FAPI” OR “DOPA” OR “DOTATOC”) AND "PET”

“clear cell” AND (“FDG” OR “PSMA” OR “FAPI” OR “DOPA” OR “DOTATOC”) AND "PET”

6 results

Renal pelvis and ureteral cancer

01/04/2023

“renal pelvis” AND (“FDG” OR “PSMA” OR “FAPI” OR “DOPA” OR “DOTATOC”) AND "PET”

“renal pelvic” AND (“FDG” OR “PSMA” OR “FAPI” OR “DOPA” OR “DOTATOC”) AND "PET”

“ureter” AND (“FDG” OR “PSMA” OR “FAPI” OR “DOPA” OR “DOTATOC”) AND "PET”

0 results

Bladder cancer

01/04/2023

“urothelial” AND (“FDG” OR “PSMA” OR “FAPI” OR “DOPA” OR “DOTATOC”) AND "PET”

“transitional cell” AND (“FDG” OR “PSMA” OR “FAPI” OR “DOPA” OR “DOTATOC”) AND "PET”

“bladder” AND (“FDG” OR “PSMA” OR “FAPI” OR “DOPA” OR “DOTATOC”) AND "PET”

6 results

Ovarian cancer

01/04/2023

“ovary” AND (“FDG” OR “PSMA” OR “FAPI” OR “DOPA” OR “DOTATOC”) AND "PET”

“ovarian” AND (“FDG” OR “PSMA” OR “FAPI” OR “DOPA” OR “DOTATOC”) AND "PET”

8 results

Uterine cancer

01/04/2023

“uterus” AND (“FDG” OR “PSMA” OR “FAPI” OR “DOPA” OR “DOTATOC”) AND "PET”

“uterine” AND (“FDG” OR “PSMA” OR “FAPI” OR “DOPA” OR “DOTATOC”) AND "PET”

“endometrial” AND (“FDG” OR “PSMA” OR “FAPI” OR “DOPA” OR “DOTATOC”) AND "PET”

“endometrium” AND (“FDG” OR “PSMA” OR “FAPI” OR “DOPA” OR “DOTATOC”) AND "PET”

20 results

Cervical cancer

01/04/2023

“cervix” AND (“FDG” OR “PSMA” OR “FAPI” OR “DOPA” OR “DOTATOC”) AND "PET”

“cervical” AND (“FDG” OR “PSMA” OR “FAPI” OR “DOPA” OR “DOTATOC”) AND "PET”

21 results

Testicular cancer

01/04/2023

“testis” AND (“FDG” OR “PSMA” OR “FAPI” OR “DOPA” OR “DOTATOC”) AND "PET”

“testicular” AND (“FDG” OR “PSMA” OR “FAPI” OR “DOPA” OR “DOTATOC”) AND "PET”

“seminoma” AND (“FDG” OR “PSMA” OR “FAPI” OR “DOPA” OR “DOTATOC”) AND "PET”

3 results

Melanoma

01/04/2023

“melanoma” AND (“FDG” OR “PSMA” OR “FAPI” OR “DOPA” OR “DOTATOC”) AND "PET”

8 results

Skin squamous cell carcinoma

01/04/2023

“skin” AND “squamous cell” AND (“FDG” OR “PSMA” OR “FAPI” OR “DOPA” OR “DOTATOC”) AND "PET”

0 results

Skin basal cell carcinoma

01/04/2023

“basal cell” AND (“FDG” OR “PSMA” OR “FAPI” OR “DOPA” OR “DOTATOC”) AND "PET”

0 results

Prostate cancer

04/06/2023

“prostate” AND (“FDG” OR “PSMA” OR “FAPI” OR “DOPA” OR “DOTATOC”) AND "PET”

40 results

| **Supplementary Table 2. Results summary of the selected meta-analyses on the diagnostic performance of PET radiopharmaceuticals in solid tumours.** | | | | | | | | | | | | |
| --- | --- | --- | --- | --- | --- | --- | --- | --- | --- | --- | --- | --- |
| **First Author, Year, Reference** | **Cancer** | **Clinical setting** | **Radio**  **pharmaceutical** | **Imaging modality** (PET/CT; PET/MR; PET/CT + PET/MR) | **Analysis** | **Number of included patients** | **AUC** | **95%CIs** | **Sensitivity** | **95%CIs** | **Specificity** | **95%CIs** |
| Xiao Y, 2015, [11] | HNSCC | Diagnosis | [^18^F]FDG | PET/MRI | Per-lesion | 421 | 0.9555 | SE 0.0130 | 0.91 | 0.89-0.93 | 0.63 | 0.60-0.66 |
| Shen G, 2014, [12] | HNSCC | N staging | [^18^F]FDG | PET/CT | Per-lesion | 731 (2,396 tot) | 0.9734 | NA | 0.90 | 0.87-0.93 | 0.92 | 0.89-0.95 |
| Chang MC, 2013, [13] | HNSCC | M staging | [^18^F]FDG | PET and PET/CT | Per-patient | 172 with M (1,069 tot) | 0.97 | SE 0.0057 | 0.83 | 0.77-0.88 | 0.97 | 0.95-0.98 |
| Deppen SA, 2014, [15] | NSCLC | Diagnosis (dd infections) | [^18^F]FDG | PET and PET/CT | Per-lesion | 8,511 nodules | 0.90 | 0.87-0.92 | 0.89 | 0.86-0.91 | 0.75 | 0.71-0.79 |
| Zhang L, 2013, [16] | NSCLC | Diagnosis (dd benign lesions) | [^18^F]FDG | Dual point PET/CT | Per-patient | 415 | 0.8244 | 0.0343 | 0.79 | 0.74-0.84 | 0.73 | 0.65-0.79 |
| Wang Z, 2015, [17] | NSCLC | Diagnosis (dd benign lesions) | [^18^F]FLT | PET and PET/CT | Per-lesion | 548 | 0.87 | NA | 0.80 | 0.74-0.85 | 0.82 | 0.74-0.88 |
| Shen G, 2017, [18] | NSCLC | N staging | [^18^F]FDG | PET/CT | Per-lesion | 4,681 (5,483 tot) | 0.88 | 0.84-0.90 | 0.65 | 0.63-0.67 | 0.93 | 0.93-0.94 |
| Wu Y, 2013, [19] | NSCLC | M staging | [^18^F]FDG | PET/CT | Per-patient | 360 (3,248 tot) | 0.96 | 0.94-0.97 | 0.77 | 0.47-0.93 | 0.95 | 0.92-0.97 |
| Qu X, 2012, [20] | NSCLC | Bone M staging | [^18^F]FDG | PET/CT | Per-patient | 1,855 pts and 1,845 bone lesions (2,940 tot) | 0.9889 | NA | 0.92 | 0.88-0.95 | 0.98 | 0.97-0.98 |
| " | NSCLC | Bone M staging | [^18^F]FDG | PET | Per-patient | 840 pts and 1,211 bone lesions) | 0.9603 | NA | 0.87 | 0.81-0.92 | 0.94 | 0.92-0.96 |
| Lu YY, 2014, [21] | SCLC | M staging | [^18^F]FDG | PET and PET/CT | Per-patient | 369 | 0.9842 | SE 0.0066 | 0.975 | 0.942-0.992 | 0.982 | 0.949-0.996 |
| Jiang Y, 2019, [22] | Lung NET | Diagnosis | [^18^F]FDG | PET/CT | Per-patient | 341 (352 tot) | NA | NA | 0.71 | 0.66-0.76 | NA | NA |
| " | Lung NET | Diagnosis | DOTA-SSR | PET/CT | Per-patient | 107 (352 tot) | NA | NA | 0.9 | 0.82-0.95 | NA | NA |
| NA | Mesothelioma |  |  |  |  |  |  |  |  |  |  |  |
| NA | Thymoma |  |  |  |  |  |  |  |  |  |  |  |
| Qichang W, 2020, [24] | DTC | Diagnosis (nodules with indeterminate cytology) | [^18^F]FDG | PET | Per-patient and per-lesion mix | 214 (634 tot) | NA | NA | 0.95 | 0.82-0.99 | 0.58 | 0.51-0.66 |
| " | DTC | " | " | PET/CT | Per-patient and per-lesion mix | 420 (634 tot) | NA | NA | 0.73 | 0.64–0.81 | 0.56 | 0.51–0.62 |
| Kim DH, 2020, [25] | DTC | N staging | [^18^F]FDG | PET/CT | Per-patient | 759 | 0.84 | SE 0.0808 | 0.3 | 0.26-0.35 | 0.94 | 0.92-0.95 |
| NA | MTC |  |  |  |  |  |  |  |  |  |  |  |
| Ruan D, 2023, [27] | Breast | Diagnosis | [^18^F]FDG | PET/MRI | Per-lesion | NA (1,723 tot) | 0.96 | 0.91-0.99 | 0.95 | 0.90-0.99 | 0.91 | 0.84-0.96 |
| " | Breast | " | " | " | Per-patient | NA (1,723 tot) | 1.00 | 0.52-1.00 | 0.97 | 0.87-1.00 | 0.97 | 0.92-0.99 |
| Lu XR, 2021, [28] | Breast | N staging | [^18^F]FDG | PET/MRI | Per-lesion | 216 (666 tot) | 0.96 | 0.94-0.97 | 0.94 | 0.83-0.98 | 0.90 | 0.81-0.95 |
| Liang X, 2017, [29] | Breast | N staging | [^18^F]FDG | PET/CT | Per-lesion | 943 | 0.89 | 0.84-0.93 | 0.64 | 0.59-0.69 | 0.93 | 0.90-0.95 |
| Ruan D, 2023, [27] | Breast | M staging | [^18^F]FDG | PET/MRI | Per-lesion | 232 (1,723 tot) | 0.95 | 0.85-1.00 | 0.94 | 0.81-0.99 | 0.92 | 0.84-0.97 |
| " | Breast | " | " | " | Per-patient | 480 (1,723 tot) | 0.97 | 0.06-1.00 | 0.98 | 0.94-1.00 | 0.96 | 0.93-0.98 |
| Shie P, 2008, [30] | Breast | Bone M staging | [^18^F]FDG | PET | Per-lesion | 184 (301 tot) | 0.09 | NA | 0.69 | NA | 0.98 | NA |
| " | Breast | " | " | " | Per-patient | 1,297 lesions | 0.08 | NA | 0.78 | NA | 0.79 | NA |
| Hu J, 2018, [32] | Oesophageal | N staging | [^18^F]FDG | PET/CT | Per-patient | 761 (1,142 tot) | 0.73 | 0.69-0.76 | 0.54 | 0.42–0.65 | 0.82 | 0.71–0.89 |
| " | Oesophageal | " | " | " | Per-station | 593 (1,142 tot) | 0.96 | 0.94-0.97 | 0.63 | 0.38–0.83 | 0.96 | 0.94–0.98 |
| van Vliet EP, 2009, [33] | Oesophageal | M staging | [^18^F]FDG | PET | Per-patient | 475 | NA | NA | 0.71 | 0.62 – 0.79 | 0.93 | 0.89 – 0.97 |
| Wang Y, 2023, [34] | Gastric | T detection | [68Ga]Ga-FAPI-04 | PET/CT and PET/MRI | Per-patient | 122 (148 tot) | NA | NA | 1.00 | NA | NA | NA |
| " | Gastric | " | FDG | " | Per-patient | " | NA | NA | 0.8443 | NA | NA | NA |
| " | Gastric | N staging | [68Ga]Ga-FAPI-04 | " | Per-patient | 61 (148 tot) | NA | NA | 0.8197 | NA | NA | NA |
| " | Gastric | " | [^18^F]FDG | " | Per-patient | " | NA | NA | 0.6721 | NA | NA | NA |
| " | Gastric | Peritoneal M staging | [68Ga]Ga-FAPI-04 | " | Per-patient | 38 (148 tot) | NA | NA | 1.00 | NA | NA | NA |
| " | Gastric | " | [^18^F]FDG | " | Per-patient | " | NA | NA | 0.4474 | NA | NA | NA |
| Mirshahvalad SA, 2022, [35] | Colorectal | Diagnosis | [^18^F]FDG | PET/MRI | Per-patient | 205 (1,534 tot) | NA | NA | 0.95 | 0.31–1.00 | 0.79 | 0.52-0.93 |
| Son GM, 2019, [36] | Colorectal | Diagnosis (incidental colorectal findings) | [^18^F]FDG | PET/CT | Per-patient | 1,451 | 0.91 | 0.88-0.93 | 0.85 | 0.78-0.90 | 0.87 | 0.77-0.94 |
| Mirshahvalad SA, 2022, [35] | Colorectal | N staging | [^18^F]FDG | PET/MRI | Per-patient | 114 (1,534 tot) | NA | NA | 0.81 | 0.65-0.91 | 0.88 | 0.76-0.94 |
| Dahmarde H, 2020, [37] | Colorectal | N staging | [^18^F]FDG | PET/CT | Per-patient | 1,460 | NA | NA | 0.66 | 0.63-0.69 | 0.76 | 0.73-0.78 |
| Mirshahvalad SA, 2022, [35] | Colorectal | M staging | [^18^F]FDG | PET/MRI | Per-patient | 297 (1,534 tot) | NA | NA | 0.97 | 0.86-0.99 | 0.93 | 0.85-0.97 |
| Maffione AM, 2015, [38] | Colorectal | Liver M staging | [^18^F]FDG | PET and PET/CT | Per-patient | 270 (484 tot) | 0.9683 | SE 0.0154 | 0.93 | 0.88-0.96 | 0.93 | 0.84-0.98 |
| Jones M, 2015, [39] | Anal | T detection | [^18^F]FDG | PET and PET/CT | Per-patient | 348 (494 tot) | NA | NA | 0.99 | 0.96-1.00 | NA | NA |
| Caldarella C, 2014, [40] | Anal | N staging | [^18^F]FDG | PET/CT | Per-lesion | 264 | 0.8325 | SE 0.0363 | 0.56 | 0.45-0.67 | 0.90 | 0.86-0.93 |
| Tang S, 2011, [41] | Pancreatic | Diagnosis | [^18^F]FDG | PET/CT | Per-patient |  | 0.94 | 0.0241 | 0.9 | 0.85-0.94 | 0.8 | 0.73-0.86 |
| Wang Z, 2013, [42] | Pancreatic | N staging | [^18^F]FDG | PET | Per-patient | 101 (1,582 tot) | NA | NA | 0.64 | 0.50-0.76 | 0.81 | 0.25-0.85 |
| Wang Z, 2013, [42] | Pancreatic | Liver M staging | [^18^F]FDG | PET and PET/CT | Per-patient | 316 (1,582 tot) | NA | NA | 0.67 | 0.52-0.79 | 0.96 | 0.89-0.98 |
| Yi Yanga,  2021, [23] | Insulinoma | Diagnosis | GLP-1R -targeting | PET/CT | Per-patient | 708 | NA | NA | 0.79 | 0.54–0.92 | 0.84 | 0.20–0.99 |
| Lin CY, 2012, [43] | HCC | M staging | [^18^F]FDG | PET and PET/CT | Per-patient | 239 (348 tot) | 0.8832 | SE 0.0774 | 0.76 | 0.68-0.83 | 0.98 | 0.92-0.99 |
| Annunziata S, 2014, [46] | Colangiocarcinoma | T staging | [^18^F]FDG | PET and PET/CT | Per-patient | 1232 for sensitivity and 768 for specificity (1,232 tot) | 0.89 | NA | 0.81 | 0.78-0.83 | 0.82 | 0.75-0.87 |
| Hu JH, 2018, [47] | Colangiocarcinoma | N staging | [^18^F]FDG | PET and PET/CT | Per-patient | 509 (1,037 tot) | 0.858 | 0.0729 | 0.516 | 0.436-0.595 | 0.914 | 0.873-0.945 |
| Huang X, 2020, [48] | Colangiocarcinoma | M staging | [^18^F]FDG | PET/CT | Per-patient | 269 (731 tot only PET – 1,626 tot PET and MRI) | 0.90 | 0.87-0.92 | 0.56 | 0.42–0.69 | 0.95 | 0.91–0.97 |
| Annunziata S, 2015, [45] | Gallbladder | T staging | [^18^F]FDG | PET and PET/CT | Per-patient | 292 for sensitivity and 218 for specificity (495 tot) | 0.8787 | SE 0.0278 | 0.87 | 0.82-0.92 | 0.78 | 0.68-0.86 |
| Liu X, 2020, [49] | GEP NET | Diagnosis | [^18^F]FDG | PET and PET/CT | Per-patient | 769 (3,401 tot) | 0.94 | 0.92-0.96 | 0.7 | 0.41-0.89 | 0.97 | 0.70-1.00 |
| " | GEP NET | " | DOTA-SSTR | " | Per-patient | 3035 (3,401 tot) | 0.96 | 0.94-0.98 | 0.92 | 0.89-0.95 | 0.91 | 0.83-0.95 |
| " | GEP NET | " | " | " | Per-lesion | 5,793 lesions | 0.98 | 0.96-0.99 | 0.95 | 0.86-0.98 | 0.93 | 0.83-0.97 |
| Piccardo A, 2021, [50] | GEP NET | Diagnosis | [^18^F]DOPA | PET and PET/CT | Per-patient | 112 | NA | NA | 0.83 | 0.70-0.92 | NA | NA |
| " | GEP NET | " | " | " | Per-lesion | 1,308 lesions | NA | NA | 0.95 | 0.89-0.99 | NA | NA |
| Han S, 2019, [52] | Pheochromocytoma Paraganglioma | T detection | [68Ga]Ga-DOTA-peptides | PET | Per-lesion | 215 | NA | NA | 0.93 | 0.91-0.95 | NA | NA |
| Kan Y, 2018, [53] | Pheochromocytoma Paraganglioma | N&M staging | [^18^F]FDG | PET/CT | Per-lesion | 738 | NA | NA | 0.83 | 0.68-0.92 | 0.74 | 0.14-0.98 |
| Yin Q, 2022, [54] | RCC | Diagnosis | [^18^F]FDG | PET | Per-patient | NA (2,545 tot) | 0.88 | 0.85-0.90 | 0.83 | 0.64-0.93 | 0.86 | 0.75-0.92 |
| " | RCC | " | " | PET/CT | Per-patient | NA (2,545 tot) | 0.94 | 0.92-0.96 | 0.89 | 0.72-0.96 | 0.88 | 0.76-0.95 |
| Wang HY, 2012, [55] | RCC | N&M staging | [^18^F]FDG | PET | Per-patient | 272 (507 tot) | 0.8979 | SE 0.0309 | 0.79 | 0.71-0.86 | 0.90 | 0.82-0.95 |
| " | RCC | " | " | " | Per-lesion | 78 (507 tot) and 113 lesions | 0.9388 | SE 0.0742 | 0.84 | 0.75-0.91 | 0.91 | 0.72-0.99 |
| Wang N, 2014, [57] | Bladder | T detection | [^18^F]FDG | PET and PET/CT | Per-lesion | 143 | 0.8574 | SE 0.0704 | 0.8 | 0.71–0.87 | 0.84 | 0.69-0.93 |
| Soubra A, 2016, [58] | Bladder | N staging | [^18^F]FDG | PET/CT | Per-lesion | 384 | NA | NA | 0.56 | 0.47 -0.65 | 0.95 | 0.92-0.98 |
| Hu X, 2021, [59] | Ovarium | Diagnosis | [^18^F]FDG | PET/CT | Per-patient | 892 (3,730 tot) | 0.95 | 0.93-0.96 | 0.94 | 0.87–0.97 | 0.86 | 0.79–0.91 |
| Yuan Y, 2012, [60] | Ovarium | N staging | [^18^F]FDG | PET and PET/CT | Per-patient | 882 | 0.9563 | SE 0.0203 | 0.732 | 0.68–0.78 | 0.967 | 0.96–0.98 |
| Han S, 2018, [61] | Ovarium | M staging | [^18^F]FDG | PET and PET/CT | Per-site | 594 | 0.89 | 0.86-0.91 | 0.72 | 0.61-0.81 | 0.93 | 0.85-0.97 |
| Kakhki VR, 2013, [62] | Endometrial | Diagnosis | [^18^F]FDG | PET and PET/CT | NA | NA (807 tot) | 0.9039 | SE 0.0298 | 0.818 | 0.779-0.853 | 0.898 | 0.792-0.962 |
| Reijnen C, 2019, [63] | Endometrial | N staging | [^18^F]FDG | PET/CT | Per-patient | 1,704 (18,205 tot) | 0.773 | NA | 0.67 | 0.61–0.73 | 0.91 | 0.87–0.94 |
| Kakhki VR, 2013, [62] | Endometrial | M staging | [^18^F]FDG | PET and PET/CT | NA | NA (807 tot) | 0.9743 | SE 0.0134 | 0.96 | 0.85-0.99 | 0.96 | 0.93-0.97 |
| Liu B, 2017, [64] | Cervical | N staging | [^18^F]FDG | PET and PET/CT | Per-lesion | NA | 0.90 | 0.88–0.92 | 0.55 | 0.44–0.65 | 0.98 | 0.96–0.99 |
| " | Cervical | " | " | " | Per-patient | NA | 0.95 | 0.93–0.97 | 0.76 | 0.60–0.87 | 0.94 | 0.91–0.96 |
| Triumbari EKA, 2021, [65] | Vulvar | N staging | [^18^F]FDG | PET and PET/CT | Per-patient | 72 | NA | NA | 0.7 | 0.44-0.95 | 0.9 | 0.76-1.00 |
| " | Vulvar | " | " | " | Per-region | 245 groins | NA | NA | 0.76 | 0.57-0.94 | 0.88 | 0.82-0.94 |
| " | Vulvar | " | " | " | Per-lesion | NA (2,104 tot) | NA | NA | 0.615 | 0.406-0.798 | 0.909 | 0.80-0.97 |
| Satapathy S, 2021, [66] | Prostate | T detection | [^68^Ga]Ga-PSMA-11 | PET/CT | Per-patient |  | 0.91 | 0.88–0.93 | 0.97 | 0.90-0.99 | 0.66 | 0.52-0.78 |
| Huang YT, 2022, [67] | Prostate | T detection | [^18^F]F-PSMA-1007 | PET/CT | Per-lesion | 458 (712 tot) | NA | NA | 0.96 | 0.94-0.98 | NA | NA |
| Yadav D, 2022, [68]  . | Prostate | T detection | Fluciclovine | PET/CT | Per-lesion | 104 (647 tot) | NA | NA | 0.85 | 0.73-0.92 | 0.77 | 0.60-0.88 |
| Zhou J, 2022, [69] | Prostate | N staging | [^68^Ga]Ga-PSMA | PET/CT | Per-patient | 1,567 | 0.95 | 0.93-0.97 | 0.61 | 0.39-0.79 | 0.96 | 0.92-0.98 |
| " | Prostate | " | " | " | Per-lesion | 1567 | 0.99 | 0.97-0.99 | 0.74 | 0.50-0.89 | 0.99 | 0.98-1.00 |
| Evangelista L, 2021, [70] | Prostate | N staging | PSMA-targeting radiopharmaceuticals | PET/MR | Per-patient | NA (2,104 tot) | NA | NA | 0.667 | 0.498-0.809 | 0.934 | 0.875-0.971 |
| " | Prostate | " | " | " | Per-lesion | NA (2,104 tot) | NA | NA | 0.643 | 0.441-0.814 | 0.974 | 0.91-0.997 |
| Zhao R, 2021, [71] | Prostate | Bone M staging | [^68^Ga]Ga-PSMA | PET/CT | Per-patient | 318 | 0.9973 | SE 0.0046 | 0.97 | 0.92-0.99 | 1.00 | 0.98-1.00 |
| Zhou J, 2019, [72] | Prostate | Bone M staging | [^11^C]- and [^18^F]-Choline | PET/CT | Per-patient | 638 (1,610 tot) | 0.99 | 0.98-1 | 0.87 | 0.80-0.92 | 0.99 | 0,96-1 |
| " | Prostate | " | [^18^F]NaF | " | Per-patient | 379 (1,610 tot) | 0.99 | 0.98-1 | 0.96 | 0.87.0.99 | 0.97 | 0,90-0,99 |
| Zhao JY, 2014, [73] | Testicular | Diagnosis | [^18^F]FDG | PET/CT | Per-patient | 807 | 0.8752 | SE 0.0344 | 0.75 | 0.70-0.80 | 0.87 | 0.84-0.89 |
| Sadeghi R, 2012, [74] | Penile | N staging | [^18^F]FDG | PET/CT | Per-region | 115 (213 lesions) | 0.9 | SE 0.0515 | 0.81 | 0.70-0.89 | 0.92 | 0.87-0.96 |
| Rodriguez Rivera AM, 2014, [75] | Melanoma | N staging | [^18^F]FDG | PET/CT | Per-patient | 623 | 0.94 | 0.92-0.96 | 0.89 | 0.65-0.97 | 0.88 | 0.77-0.94 |
| Shim SR, 2022, [76] | MCC | N staging | [^18^F]FDG | PET and PET/CT | Per-patient | 259 | 0.97 | 0.95-0.98 | 0.91 | 0.85-0.95 | 0.93 | 0.86-0.97 |
| Seth N, 2022, [77] | Ewing Sarcoma | Diagnosis | [^18^F]FDG | PET and PET/CT | Per-lesion | 218 (735 tot) | NA | NA | 0.967 | 0.922-0.986 | 0.683 | 0.44-0.856 |
| " | Ewing Sarcoma | N staging | " | " | Per-patient | 170 (735 tot) | NA | NA | 0.793 | 0.587-0.912 | 0.979 | 0.935-0.993 |
| " | Ewing Sarcoma | Lung M staging | " | " | Per-patient | 219 (735 tot) | NA | NA | 0.761 | 0.614-0.865 | 0.924 | 0.863-0.959 |
| Huang T, 2018, [78] | Ewing Sarcoma | Bone M staging | [^18^F]FDG | PET and PET/CT | Per-patient | 246 (524 tot) | 0.9859 | SE 0.0085 | 0.91 | 0.80-0.97 | 0.98 | 0.94-0.99 |
| Zhang Q, 2020, [80] | Chondrosarcoma | Diagnosis | [^18^F]FDG | PET/CT | Per-patient | 270 (375 tot) | 0.92 | 0.89-0.94 | 0.94 | 0.86-0.97 | 0.89 | 0.82-0.93 |
| AUC – area under the curve; CI – confidence interval; CT – computed tomography; [^18^F]FDG – 2-deoxy-2-[^18^F]fluoro-D-glucose; DOTA-peptides – somatostatin receptor targeting PET radiopharmaceuticals with DOTA; DTC – differentiated thyroid cancer; FAPI – fibroblast activation protein inhibitors; GEP NET – gastroenteropancreatic neuroendocrine tumours; GLP-1R - Glucagon-Like Peptide-1 Receptor; HCC – hepatocellular carcinoma; HNSCC – head and neck squamous cell carcinoma; MTC – medullary thyroid cancer; NA – not available; NET – neuroendocrine tumours; NSCLC – non small cell lung cancer; MCC – Merkel cell carcinoma; MRI – magnetic resonance imaging; N staging – lymph-nodal staging; M staging – distant metastases staging; PET – positron emission tomography; PSMA – prostate specific membrane antigen; RCC – renal clear-cell carcinoma; SCLC – small cell lung cancer | | | | | | | | | | | | |
